# LncRNA-NONHSAT024778 promote the proliferation and invasion of chordoma cell by regulating miR-1290/Robo1 axis

**DOI:** 10.7150/ijbs.54091

**Published:** 2021-02-08

**Authors:** Bin Wang, Kai Zhang, Sen Meng, Xiaofeng Shao, Zhangzhe Zhou, Haiqing Mao, Ziqiang Zhu, Hao Chen, Huilin Yang, Kangwu Chen

**Affiliations:** 1 Department of Orthopedic Surgery, The First Affiliated Hospital of Soochow University, No. 188 Shizi Street, Suzhou, Jiangsu, China.; 2 Department of Orthopaedic Surgery, The Second Affiliated Hospital of Xuzhou Medical University, Xuzhou, Jiangsu, China.; 3 Jiangsu Center for the Collaboration and Innovation of Cancer Biotherapy, Cancer Institute, Xuzhou Medical University, Xuzhou, Jiangsu, China.

**Keywords:** chordoma, NONHSAT024778, miR-1290, Robo1, proliferation, invasion

## Abstract

Chordoma is a malignant bone tumor originating from the embryonic remnants of the notochord. lncRNAs act as competing endogenous RNAs (ceRNAs) and play a critical role in tumor pathology. However, the biological role of lncRNA-NONHSAT024778 and the underlying molecular mechanism in chordoma remains unknown. qRT-PCR was used to analyze the expression changes of NONHSAT024778 and miR-1290 in chordoma tissues and cell lines. Bioinformatics analysis and luciferase reporter assay were applied to detect the targeting binding effect between NONHSAT024778 and miR-1290, and between Robo1 and miR-1290. The effect of NONHSAT024778 on chordoma cell proliferation and invasion and its regulation of miR-1290 by acting as a ceRNA were also investigated. An increased NONHSAT024778 expression was correlated with a decreased miR-1290 level in chordoma tissues. NONHSAT024778 knockdown suppressed the proliferation and invasion of chordoma cells. miR-1290 restored expression rescued the carcinogenic function of NONHSAT024778. Bioinformatics analysis showed that NONHSAT024778 acted as ceRNA to regulate Robo1 via sponging miR-1290 in chordoma cells, thereby promoting chordoma cell malignant progression. *In vivo* results confirmed the anti-tumor effects of NONHSAT024778 knockdown activating miR-1290 to inhibit the oncogene Robo1. NONHSAT024778 is substantially overexpressed, whereas miR-1290 is decreased in chordoma tissue. NONHSAT024778-miR-1290-Robo1 axis plays a critical role in chordoma tumorigenesis and might be a potential predictive biomarker for the diagnosis and therapeutic target among patients with chordoma.

## Introduction

Chordoma is a rare bone tumor that is aggressive, highly recurrent, locally invasive and prone to metastasis 1. This cancer originates from transformed remnants of the embryonic notochord and has a predilection for sacrococcygeal and skull base region 2. Chordomas are poorly sensitive to conventional radiotherapy and resist chemotherapy, therefore, radical surgery with adjuvant radiotherapy is the most used treatment modality 3, 4. Complete removal of chordoma is still difficult, because patients often present with local recurrence and metastasis 5. More than 30% of patients have distant metastasis, and the overall 5-year survival rate following surgery is 47% 6, 7. Chordoma is a multistep biological process involving multiple factors, therefore, the molecular biology mechanisms underlying its initiation and progression must be uncovered to understand and develop effective therapies for this disease.

Human genome can be pervasively transcribed into short or long noncoding RNAs (ncRNAs) regarded as endogenous regulatory RNAs 8. Long noncoding RNAs (lncRNAs) are transcripts with more than 200 nucleotides (nt) in length with limited or no protein-coding capacity 9, 10. With the unprecedented research progress in their function, lncRNAs were found to play a role in the physiological and pathological processes of various diseases, especially malignancies 11, 12. MicroRNAs (miRNAs/miRs) are small, noncoding, endogenous RNAs containing 20-30 nt in length 13. miRNAs might function biologically as either tumor suppressors or oncogenes influencing the post-transcriptional gene expression involved in many cancer pathogenesis, including chordoma 14, 15. Salmena first proposed that lncRNAs could indirectly regulate their downstream genes by acting as ceRNAs and interacting with miRNAs 16. This ceRNA theory has been widely confirmed, for instance, lncRNA LINC00460 promotes colorectal cancer metastasis via miR-939-5p sponging 17, and LncRNA TUG1 contributes to human osteosarcoma tumorigenesis by sponging miR-9-5p and regulating POU2F1 expression 18.

Our previous study used miRNA microarray profiling to reveal the different miRNA expression levels between chordoma and fetal nucleus pulposus (FNP) and found that miR-1290 is the most down-regulated miRNA within chromosome 1 19. Chromosome 1 gene deletion or amplification is the most common chromosome variation in chordoma 20-22. In addition, miR-1290 has great potential as a regulator in chordoma microenvironment 19. Another of our work confirmed the different miR-1290 expression and also analyzed the clinical features and prognosis of chordoma 23. However, its association with chordoma invasion and recurrence has never been reported. Recently, we performed microarrays to detect the lncRNAs expression profile of chordoma and the control tissue FNP to determine which lncRNAs are involved in chordoma biological processes. A total of 2786 up-regulated lncRNAs and 2042 down-regulated lncRNAs were identified 24. Bioinformatics analysis revealed that novel lncRNA-NONHSAT024778 acted as a ceRNA to regulated roundabout guidance receptor 1 (Robo1) via sponging miR-1290 in chordoma.

In this study, the expression level of NONHSAT024778 and miR-1290 in chordoma was analyzed by conducting loss of-function and gain-of-function experiments in chordoma cell lines. Proliferation, migration, and invasion *in vitro* and animal tumor xenografts *in vivo* were also assessed. The molecular mechanisms underlying the relationship among NONHSAT024778, miR-1290 and Robo1 were determined to help in developing potential biomarker and therapeutic targets in the prognosis and treatment of patients with chordoma.

## Materials and Methods

### Clinical tissue specimens

This study was approved by the Ethical Committee of the First Affiliated Hospital of Soochow University. Written consent was obtained prior to subject enrollment, following informed consent at the time of acquisition. Chordoma samples were collected from 20 patients who were identified and treated by tumor resection surgeries at the Department of Orthopedics, the First Affiliated Hospital of Soochow University. None of the enrolled patients had received any chemotherapy or radiotherapy before surgical excision of the tumor lesion. FNP specimens were obtained from 10 aborted fetuses with a gestational age of 12-28 weeks in the Department of Gynecology and Obstetrics, the First Affiliated Hospital of Soochow University. Imaging and histopathological analysis confirmed the properties of chordoma and FNP tissues. All the samples were collected and stored in liquid nitrogen and homogenized in TRIzol reagent (Thermo Fisher, 15596018, USA).

### Cell culture and transfection

The chordoma cell line cells, U-CH1, were obtained from ATCC (USA). U-CH1 cells were cultured in IMDM-RPMI media (4:1) supplemented with 10% fetal bovine serum and 1% penicillin-streptomycin, and maintained in a humidified incubator with 5% CO_2_-enriched atmosphere at 37 °C.

Effective siRNA oligonucleotides that target NONHSAT024778 (si-NONHSAT024778) and scramble control were chemically synthesized by GenePharma Technology (Shanghai, China). miR-1290 mimics and negative control (NC mimic), miR-1290 inhibitor and negative control (NC inhibitor), wild NONHSAT024778 overexpression plasmids (NONHSAT024778-WT), mutant NONHSAT024778 overexpression plasmids (NONHSAT024778-Mut), NONHSAT024778 overexpression lentiviruses (Lv-NONHSAT024778), and Robo1 overexpression lentiviruses (Lv-Robo1) were also purchased from GenePharma Technology (Shanghai, China). All oligonucleotides and plasmids were individually transfected to chordoma cell lines by using a Lipofectamine RNAi MAX (Thermo Fisher, USA) in accordance with the manufacturer's instructions to obtain different expression levels of targeted gene cell models.

### RNA extract, reverse transcription, and quantitative real-time PCR (qRT-PCR)

Total RNA was extracted using TRIzol (Thermo Fisher, 15596018, USA), and cDNA was synthesized using the HiScript 1st Strand cDNA Synthesis Kit (Thermo Fisher, 12183025, 18418012, 18427088, USA). PCR reactions were conducted on ABI-7900 (Applied Biosystems, Foster City, CA) using ReverTra Ace qPCR RT Master Mix Kit (TOYOBO, FSQ-301, Japan) following the manufacturer's instructions. GAPDH and U6 were used to normalize the expression levels of NONHSAT024778 and miR-1290, respectively. Relative gene expression was calculated using the 2^-ΔΔCt^ method. Primer sequences were synthesized by GenePharma Technology (Shanghai, China) and listed in below:

- NONHSAT024778-For: TCCCCTTTGCATGTGGCTGG;
- NONHSAT024778-Rev: AAAACAGGACGGCCAGAGCC;
- ROBO1-For: GATGACCCTCGCTCACACCG;
- ROBO1-Rev: AGGATGGCCTCGTGGAGGTT.

### Cell proliferation assays

Cell counting kit-8 (CCK-8) assay was applied to measure cell proliferation. Transfected cells were seeded in a 96-well plate, and each well was added with 10 μl of CCK-8 solution (Beyotime, C0038, China) every day for 4 days, Absorbance at 450 nm was examined by a microplate reader. All experimental procedures were in accordance with the manufacturer's instructions.

### Transwell and wound healing assays

Cell migration/invasion was observed using Transwell chambers (8.0 μm, Corning, CLS3428-24EA, NY, USA.). In brief, 5×10^4^ cells were plated in the upper uncoated (for migration) and Matrigel-coated (for invasion) chambers with serum-free medium. The culture medium with 10% FBS was supplemented into the lower wells. All chambers were incubated for another 24 hours, and the non-migrated or non-invaded cells were then wiped out. The filters were fixed and stained by crystal violet staining. Five random selected fields were counted per chamber by using an inverted microscope (NIKON Corporation, Tokyo, Japan).

In wound healing assay, the cells were seeded at a density of 1×10^6^ cell/well onto six-well plates and cultured to approximately 80% confluence. A sterile 10-μl pipette tip was used to form artificial scratches for each well. The suspended cells were washed away with PBS and then cultured in a medium with 1% FBS. Cell migration distance was photographed at 0 h and 24 h under an inverted light microscope.

### Western blot

The cell lysates were prepared with RIPA buffer. Equal amounts of proteins collected from different types of cell lysates were loaded on 10-15% SDS-PAGE gels and transferred to polyvinylidene difluoride (PVDF) membranes. The membranes were incubated with primary antibodies at 4 °C overnight followed by incubation with corresponding secondary antibodies. The outcomes were examined by utilizing an electrochemiluminescence (ECL) reagent. All experiments were repeated at least three times.

### Dual luciferase reporter assay

Reporter plasmids containing the predicted microRNA binding sites, NONHSAT024778 (pisCHECK-2-NONHSAT024778-WT/Mut), and Robo1 (Robo1 3'UTR-WT and Robo1 3'UTR-Mut) were synthesized by OE Biotech Co., Ltd. (Shanghai, China). The assays were performed using a luciferase assay kit (Promega, Madison, WI, USA) in accordance with the manufacturer's protocol. The cells were first transfected with appropriate plasmids in 24-well plates, collected and lysed for luciferase assay 48h after transfection. Fluorescence signal changes in each group were evaluated by using the dual-luciferase reporter assay system (Promega) and normalized to firefly luciferase activity. All transfection experiments were independently performed in triplicates.

### Tumor xenograft model and tumor formation assay

For tumorigenicity studies, stably transfected U-CH1 cells (1×10^7^ cells/mouse, 0.2 ml) were subcutaneously injected into either side of the armpit regions of 8-week-old male Balb/c NOD nude mice purchased from Shanghai SLAC Laboratory Animal Co., Ltd (Shanghai, China). Tumor volumes were examined and calculated every 5 days using the following equation: volume =0.5 × length × width^2^. At 25 days after injection, the mice were euthanized, and the subcutaneous growth of each tumor was examined. This study was approved by the Committee on the Ethics of Animal Experiments of the First Affiliated Hospital of Soochow University.

### Bioinformatics prediction and analysis

On the basis of the previous microarray profiling results in our research 19, 24, the independent online tools miRanda (Website: http://www.microrna.org/microrna/home.do) was used to predict the binding site of NONHSAT024778 targeting miR-1290. TargetScan v7.2 (Website: http://www.targetscan.org/vert_72), microRNAorg (Website: http://www.microrna.org/microrna/home.do), and PITA (Website: http://www.genie.weizmann.ac.il/index.html) were utilized to determine the target gene Robo1 and miR-1290 binding site of Robo1 and predict the possible target genes of NONHSAT024778-miR-1290.

### Statistical analysis

All experiments were repeated in triplicate, and all data were expressed as mean ± SD. Statistical analyses were conducted using SPSS 20.0 software and GraphPad Prism 8. Differences between groups were estimated by two-tailed Student's t test or one-way ANOVA and considered significant or very significant when P-value < 0.05 or 0.01, respectively.

## Results

### NONHSAT024778 and miR-1290 expression in chordoma tissues

**Figure 1:**
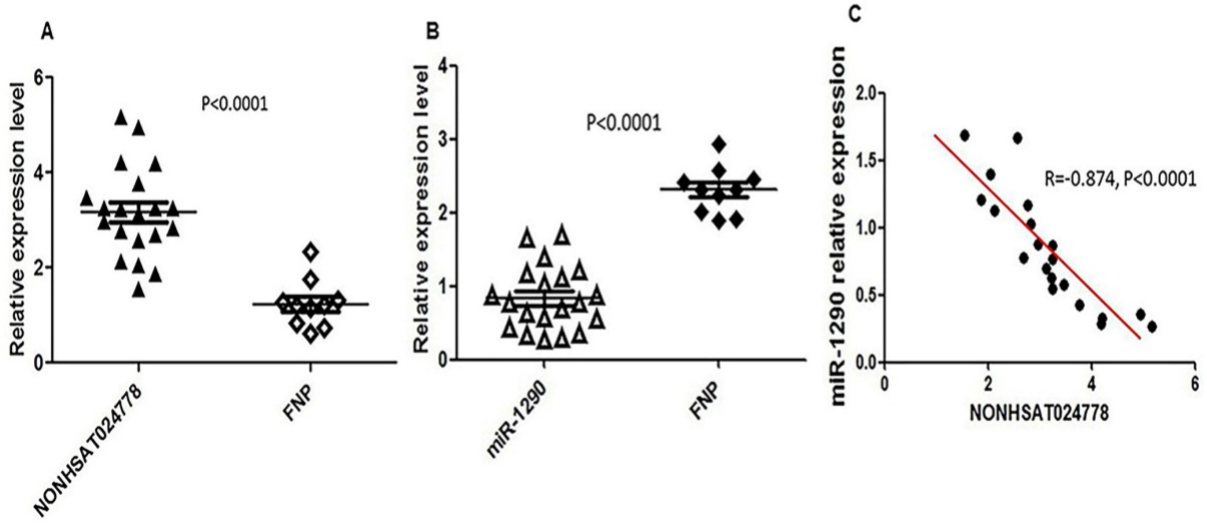
** NONHSAT024778 and miR-1290 were aberrantly expressed in chordoma tissues. (A, B)** qRT-PCR was performed to measure the mRNA levels of NONHSAT024778 and miR-1290 between chordoma and FNP tissues (*P* < 0.0001). **(C)** The correlation between NONHSAT024778 and miR-1290 was also analyzed (*P* < 0.0001).

Chordoma is thought to originate from the remnant notochord 25, which disappears by early childhood and is replaced by the nucleus pulposus in the intervertebral discs 26. Therefore, FNP tissues were selected as a control, and qRT-PCR assay was used to measure NONHSAT024778 and miR-1290 expression level in 20 clinical chordoma tissues and 10 FNP tissues. As shown, the mRNA levels of NONHSAT024778 were significantly up-regulated in the chordoma tissues compared with those in the FNP tissues (Figure 1A, P < 0.001). By contrast, miR-1290 expression was down-regulated in tumor tissues compared with that in the control (Figure 1B, P < 0.001). Statistical analysis further revealed that NONHSAT024778 transcription was inversely correlated with miR-1290 expression (Figure 1C, P < 0.001).

### NONHSAT024778 affects the proliferation, migration, and invasion of chordoma cells

**Figure 2:**
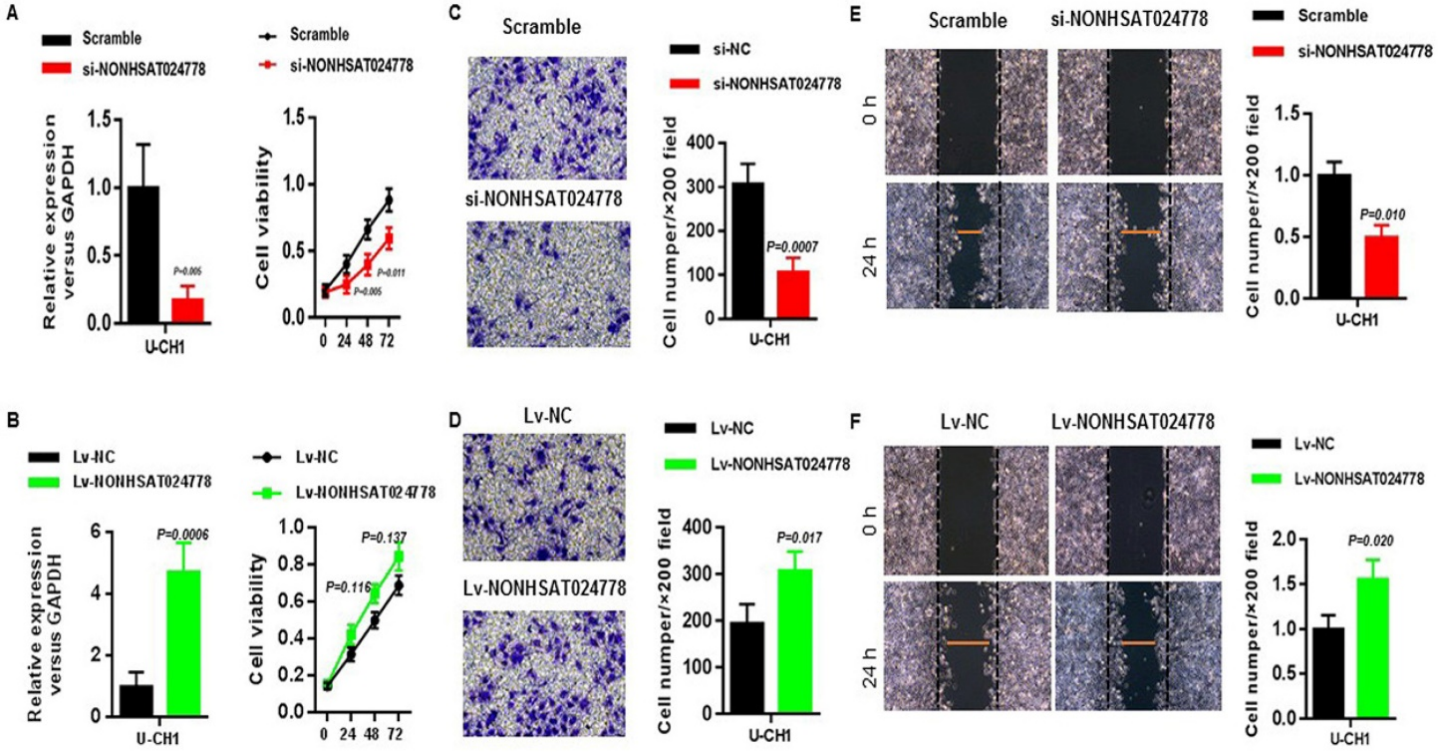
** The effects of NONHSAT024778 on chordoma cell proliferation, migration and invasion *in vitro*. (A, B)** Left, The NONHSAT024778 mRNA level was detected by qRT-PCR in si-NONHSAT024778 or Lv-NONHSAT024778 transfected U-CH1 cell (*P* < 0.05). Right, Effect of si-NONHSAT024778 or Lv-NONHSAT024778 on U-CH1 cell proliferation was assessed by Cell Counting Kit-8 assays (CCK8) (*P* < 0.05). **(C, D)** Transwell assay was used to determine cell invasion in si-NONHSAT024778 or Lv-NONHSAT024778 transfected U-CH1 cell (*P* < 0.05). **(E, F)** Wound healing assay was used to reveal cell migration in si-NONHSAT024778 or Lv-NONHSAT024778 transfected U-CH1 cell (*P* < 0.05).

Considering that NONHSAT024778 is up-regulated in chordoma tissues, U-CH1 cell line was used for the loss-of-function and gain-of-function experiments *in vitro* to explore whether NONHSAT024778 affects chordoma cell proliferation and metastatic potential (invasion and migration). CCK8 assay results showed that U-CH1 cells depleted of NONHSAT024778 displayed delayed cell growth compared with the corresponding controls (Figure 2A), whereas those with NONHSAT024778 overexpression exhibited insignificantly increased cell proliferation (Figure 2B). Transwell and wound healing assays were used to analyze the effects of NONHSAT024778 on chordoma cell invasion and migration. The transwell assays results revealed that the migration and invasion abilities of U-CH1 cells were decreased by NONHSAT024778 knockdown (Figure 2C), however, NONHSAT024778 overexpression increased cell migration and invasion (Figure 2D). In addition, wound healing assays produced the same conclusion about NONHSAT024778 positively regulating the speed of migration and invasion in U-CH1 cells (Figures 2E, 2F).

### NONHSAT024778 functions as a ceRNA and sponges miR-1290 in chordoma

**Figure 3:**
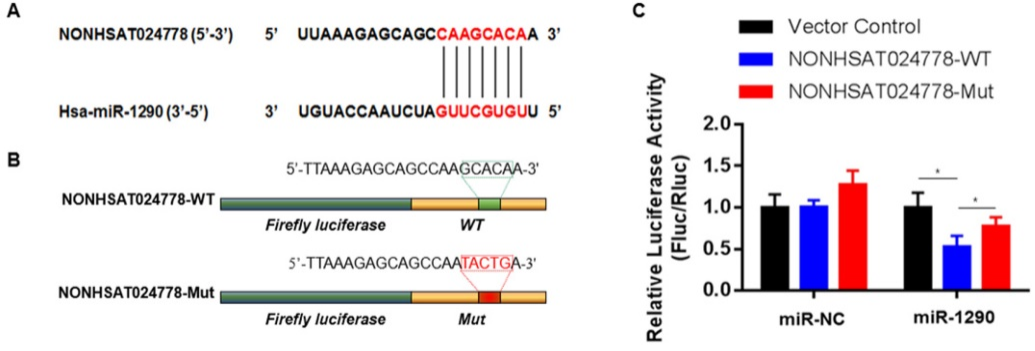
miR-1290 was a direct target of NONHSAT024778 in chordoma (*P<0.05). (A) Bioinformatic analysis identified a potential miR-1290 target site in the 3'UTR of NONHSAT024778. **(B)** A photograph displays the luciferase reporter plasmid containing wild type (WT) or mutant (Mut) NONHSAT024778. **(C)** The luciferase reporter plasmid NONHSAT024778-WT or NONHSAT024778-Mut was co-transfected into cells with miR-1290 in parallel with an NC plasmid vector.

A possible function mechanism of lncRNA is by acting as the ceRNA sponge of miRNAs 16. In the previous sections, miR-1290 was found to be significantly and negatively associated with NONHSAT024778 expression in chordoma tissues. Therefore, the correlation between NONHSAT024778 and miR-1290 was analyzed using online bioinformatics analysis programs (miRanda). The predicted binding sites of miR-1290 in NONHSAT024778 are shown (Figure 3A). Luciferase reporter assay was employed to examine whether miR-1290 directly binds to NONHSAT024778. NONHSAT024778-WT, NONHSAT024778-Mut downstream of diverse luciferase genes (Figure 3B) were cloned and co-transfected with miR-1290 mimics in U-CH1 cells. As expected, miR-1290 significantly decreased the luciferase signals of NONHSAT024778-WT reporters, but had no effect on NONHSAT024778-Mut reporters (Figure 3C). These results directly confirmed that miR-1290 could target NONHSAT024778.

### NONHSAT024778 activity is partially mediated by the negative miR-1290 regulation

**Figure 4:**
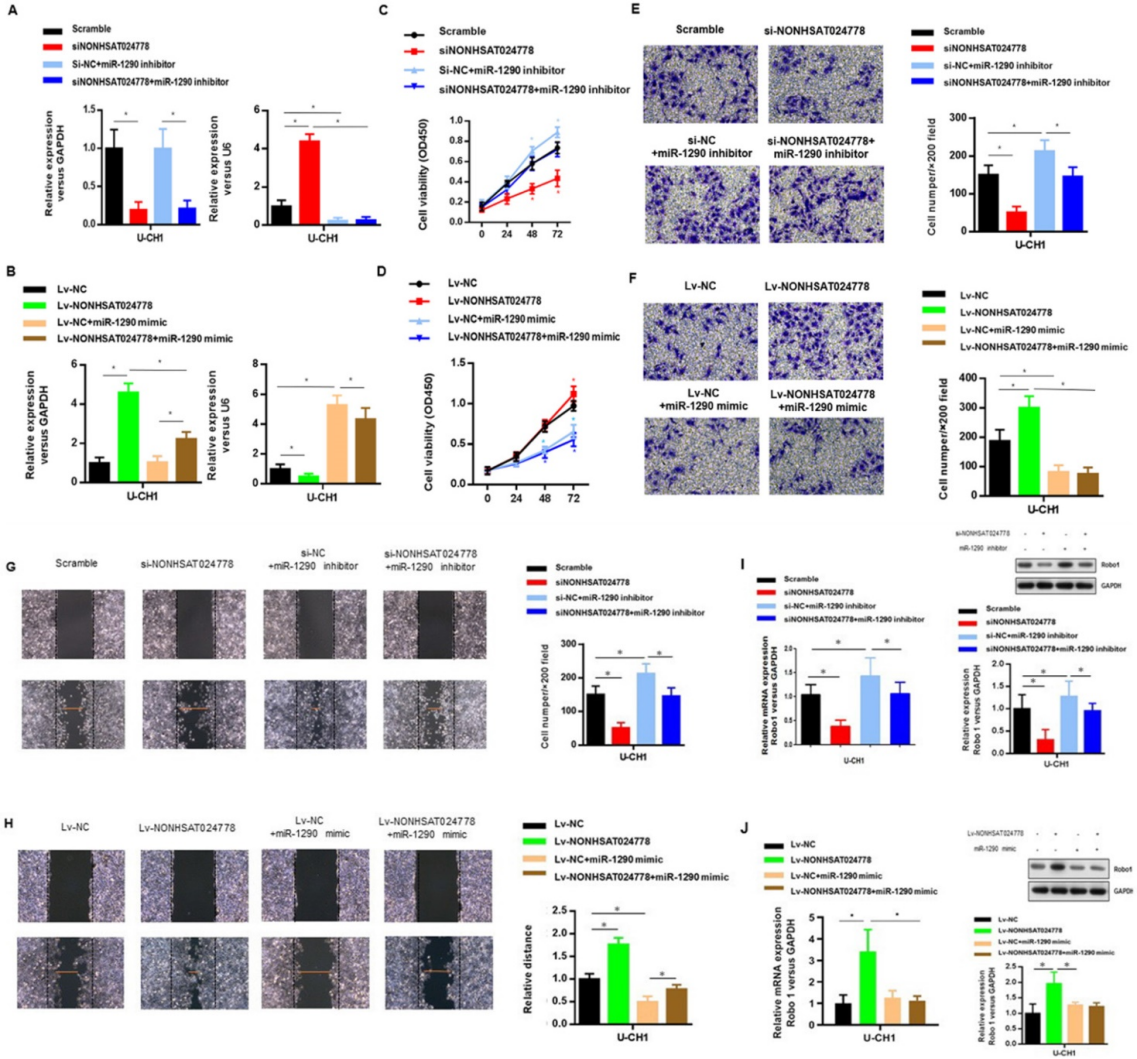
Effects of miR-1290 regulated by NONHSAT024778 on chordoma cells proliferation, migration and invasion *in vitro* (*P<0.05). (A, B) qRT-PCR was used to measure mRNA level. Left, The NONHSAT024778 mRNA level of si-NONHSAT024778+miR-1290 inhibitor or Lv-NONHSAT024778+miR-1290 mimic transfected U-CH1 cell compared with control groups. Right, the miR-1290 mRNA level of si-NONHSAT024778+miR-1290 inhibitor or Lv-NONHSAT024778+miR-1290 mimic transfected U-CH1 cell compared with control groups. **(C, D)** Growth curves of CCK8 assays for U-CH1 cells after transfected with si-NONHSAT024778+miR-1290 inhibitor or Lv-NONHSAT024778+miR-1290 mimic compared with control groups. **(E, F)** Invasion ability of U-CH1 cells after transfected with si-NONHSAT024778+miR-1290 inhibitor or Lv-NONHSAT024778+miR-1290 mimic or control vector was determined by Transwell assay. **(G, H)** Migration ability of U-CH1 cells after transfected with si-NONHSAT024778+miR-1290 inhibitor or Lv-NONHSAT024778+miR-1290 mimic or control vector was determined by wound healing assay. **(I, J)** The mRNA and protein expression of Robo1 was measured in U-CH1 cells transfected with si-NONHSAT024778+miR-1290 inhibitor or Lv-NONHSAT024778+miR-1290 mimic or control vector.

U-CH1 cells were co-transfected with siNONHSAT024778 and miR-1290 inhibitor (Figure 4A) to determine whether miR-1290 was involved in mediating the effects of NONHSAT024778 in chordoma cells. In addition, U-CH1 cells were co-transfected with Lv-NONHSAT024778 and miR-1290 mimic (Figure 4B), separately confirmed by qRT-PCR. CCK-8 assays were performed and revealed that the siNONHSAT024778-mediated inhibition of cell proliferation was partially rescued by the co-transfection with miR-1290 inhibitor (Figure 4C), Meanwhile, the Lv-NONHSAT024778-mediated promotion of cell proliferation was partially eliminated by the co-transfection with miR-1290 mimic (Figure 4D), Furthermore, transwell (Figure 4E, 4F) and wound healing assays (Figure 4G, 4H) were conducted in the same treatment condition, and produced the same conclusion about the coordinated relationship of NONHSAT024778 and miR-1290 affecting cell migration and invasion. The mRNA and protein levels of Robo1 presented similar trends in U-CH1 cells following si-NONHSAT024778+miR-1290 inhibitor or Lv-NONHSAT024778+miR-1290 mimic transfection (Figure 4I, 4J). All these results suggested that NONHSAT024778 promoted cell proliferation, migration and invasion by suppressing miR-1290 activity to some extent.

### Robo1 is a miR-1290 target gene and is indirectly regulated by NONHSAT024778

**Figure 5:**
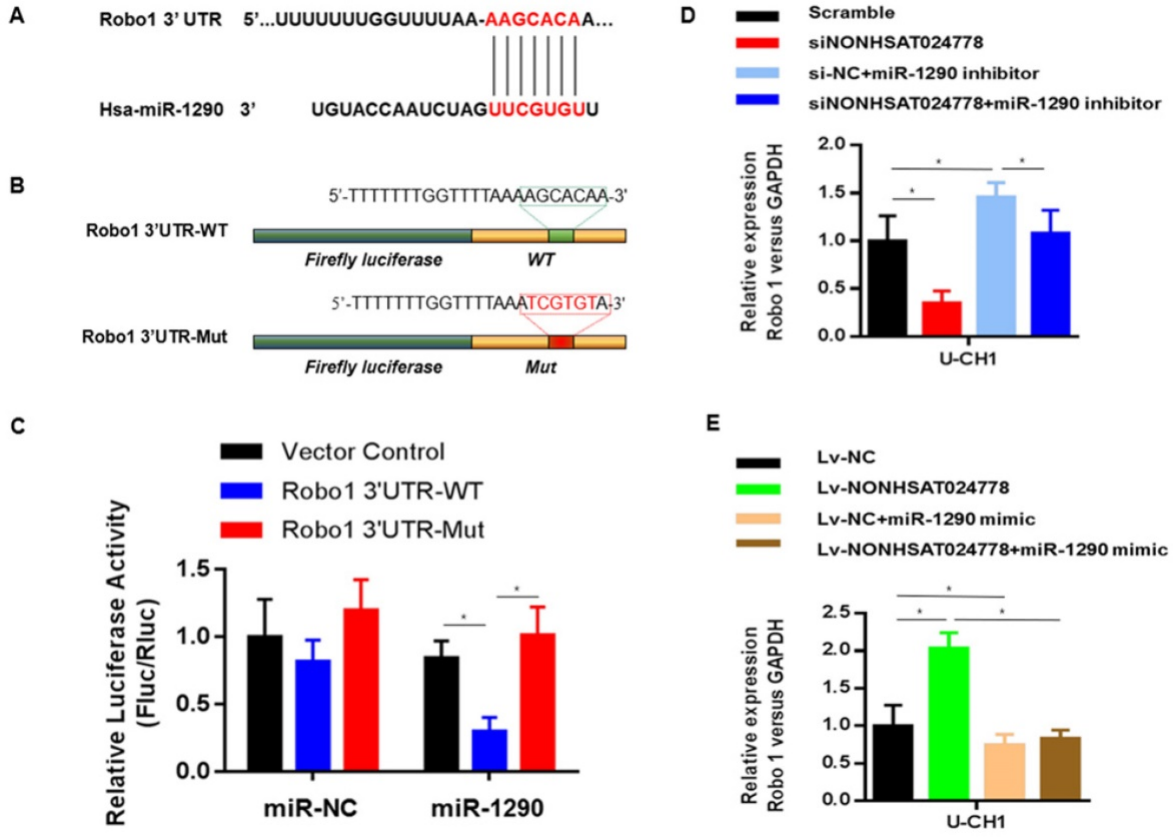
Robo1 is a target of miR-1290 and is regulated by NONHSAT024778/miR-1290(*P<0.05). (A) Bioinformatic analysis identified a potential miR-1290 target site in the 3'UTR of Robo1. **(B)** A photograph displays the luciferase reporter plasmid containing wild type (WT) or mutant (Mut) Robo1. **(C)** The luciferase reporter plasmid Robo1-3'UTR-WT or Robo1-3'UTR-Mut was co-transfected into cells with miR-1290 in parallel with an empty plasmid vector. **(D)** Relative mRNA level of Robo1 in U-CH1 cells transfected with si-NONHSAT024778+miR-1290 inhibitor, si-NC+miR-1290 inhibitor, si-NONHSAT024778 or scramble. **(E)** Relative mRNA level of Robo1 in U-CH1 cells transfected with Lv-NONHSAT024778+miR-1290 mimic, Lv-NC+miR-1290 mimic, Lv-NONHSAT024778 or Lv-NC.

TargetScan, PITA, and miRanda were used to predict potential miR-1290 target genes and determine the ceRNA network between NONHSAT024778, miR-1290 and its targets in chordoma. Robo1 gene was found to be involved in this network. Bioinformatics analysis revealed that the 3'UTR of Robo1 contains potential binding site of miR-1290 (Figure 5A). Luciferase reporter assays were conducted as driven by the wild-type 3'UTR sequence of Robo1, which contains the predicted miR-1290 binding site (Robo1 3'UTR-WT), or the mutant constructs containing a mutation in the miR-1290 binding sites (Robo1 3'UTR-Mut) (Figure 5B). These plasmids were co-transfected into the cells together with miR-1290 mimic or negative control miRNA. The results showed that Robo1 3'UTR-WT-driven luciferase expression was significantly reduced by the co-transfection with miR-1290 mimic compared with that in the control, but this repression was abolished by the mutation of the putative miR-1290 binding site in the Robo1 3'UTR (Figure 5C).

The role of miR-1290 in the relationship between NONHSAT024778 and Robo1 was determined by examining the cells co-transfected with either siNONHSAT024778 and miR-1290 inhibitor or Lv-NONHSAT024778 and miR-1290 mimic. The suppression of Robo1 mRNA and protein levels induced by siNONHSAT024778 was effectively reversed by the miR-1290 inhibitor. Meanwhile, the increase in Robo1 mRNA and protein expression induced by Lv-NONHSAT024778 was also reversed by the miR-1290 mimic. NONHSAT024778 also influenced the Robo1 expression (Figure 5D-E). These data suggest that NONHSAT024778 indirectly modulates the level of Robo1 mRNA and protein expression by regulating miR-1290.

### NONHSAT024778/miR-1290/Robo1 affects the proliferation, migration, and invasion of chordoma cell

**Figure 6:**
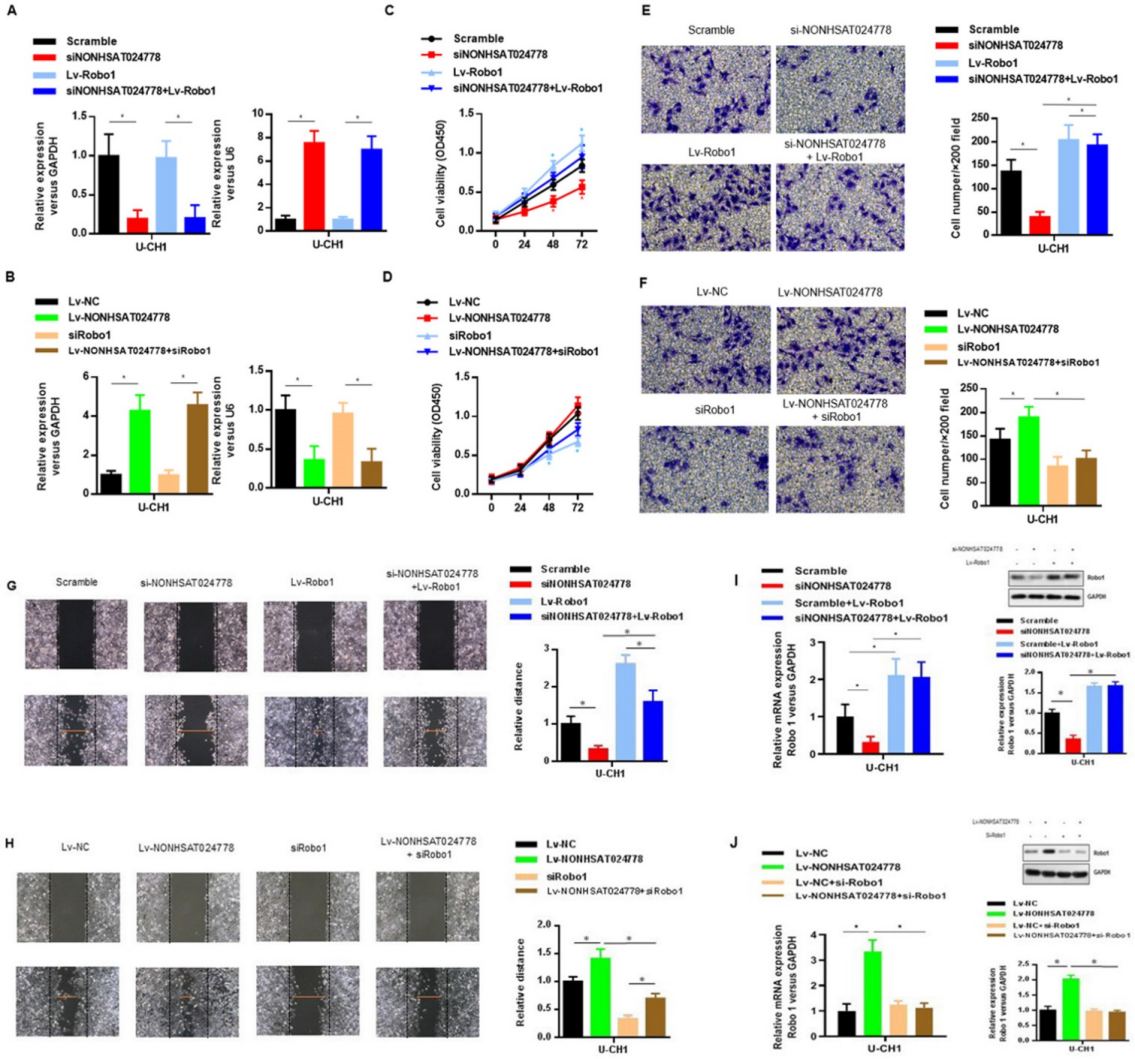
The effects of Robo1 on chordoma cells viability *in vitro* (*P<0.05). (A, B) qRT-PCR was used to measure mRNA level. Left, The NONHSAT024778 mRNA level of si-NONHSAT024778+ Lv-Robo1 or Lv-NONHSAT024778+si-Robo1 transfected U-CH1 cell in parallel with control vector. Right, The miR-1290 mRNA level of si-NONHSAT024778+Lv-Robo1 or Lv-NONHSAT024778+si-Robo1 transfected U-CH1 cell in parallel with control vector. **(C, D)** U-CH1 cell growth after transfection with si-NONHSAT024778+Lv-Robo1 or Lv-NONHSAT024778+si-Robo1 or control vector was determined by CCK8. **(E, F)** Transwell assays were used to investigate the changes in invasive ability of chordoma cells transfected with si-NONHSAT024778+Lv-Robo1 or Lv-NONHSAT024778+si-Robo1 or control vector. **(G, H)** Migration ability of U-CH1 cells after transfection with si-NONHSAT024778+Lv-Robo1, Lv-NONHSAT024778+si-Robo1 or control vector was determined by wound healing assay. **(I, J)** The mRNA and protein expression of Robo1 was measured in U-CH1 cells transfected with si-NONHSAT024778+Lv-Robo1, Lv-NONHSAT024778+si-Robo1 or control vector.

The oncogenic role of Robo1 in chordoma was investigated as follows. First, U-CH1 cells were co-transfected with si-NONHSAT024778 and Lv-Robo1 to rescue Robo1 expression caused by NONHSAT024778 knockdown (Figure 6A), U-CH1 cells were then co-transfected with Lv-NONHSAT024778 and Robo1 siRNA to knockdown the Robo1 expression induced by NONHSAT024778 overexpression as separately confirmed by qRT-PCR (Figure 6B). CCK8 assays showed that NONHSAT024778 inhibition reduced U-CH1 cell proliferation, and this phenomenon was partly reversed by the co-transfection with Lv-Robo1 (Figure 6C). The promoting effect of NONHSAT024778 for U-CH1 cell proliferation was partly reversed by the co-transfection with Robo1-targeting siRNA (Figure 6D). In addition, transwell and wound healing assays revealed that NONHSAT024778 inhibition reduced the U-CH1 cell migration and invasion, and this effect could be restored by the re-expression of Robo1 (Figures 6E, 6G). Correspondingly, the promoting function of NONHSAT024778 for U-CH1 cell migration and invasion could be weakened by re-suppressing Robo1 (Figures 6F, 6H). qRT-PCR and western blot analysis further determined that NONHSAT024778 low expression or overexpression can regulate the mRNA and protein expression level of Robo1 (Figure 6I, 6J).

### NONHSAT024778/miR-1290/Robo1 affects the tumorigenesis of chordoma cells *in vivo*

**Figure 7:**
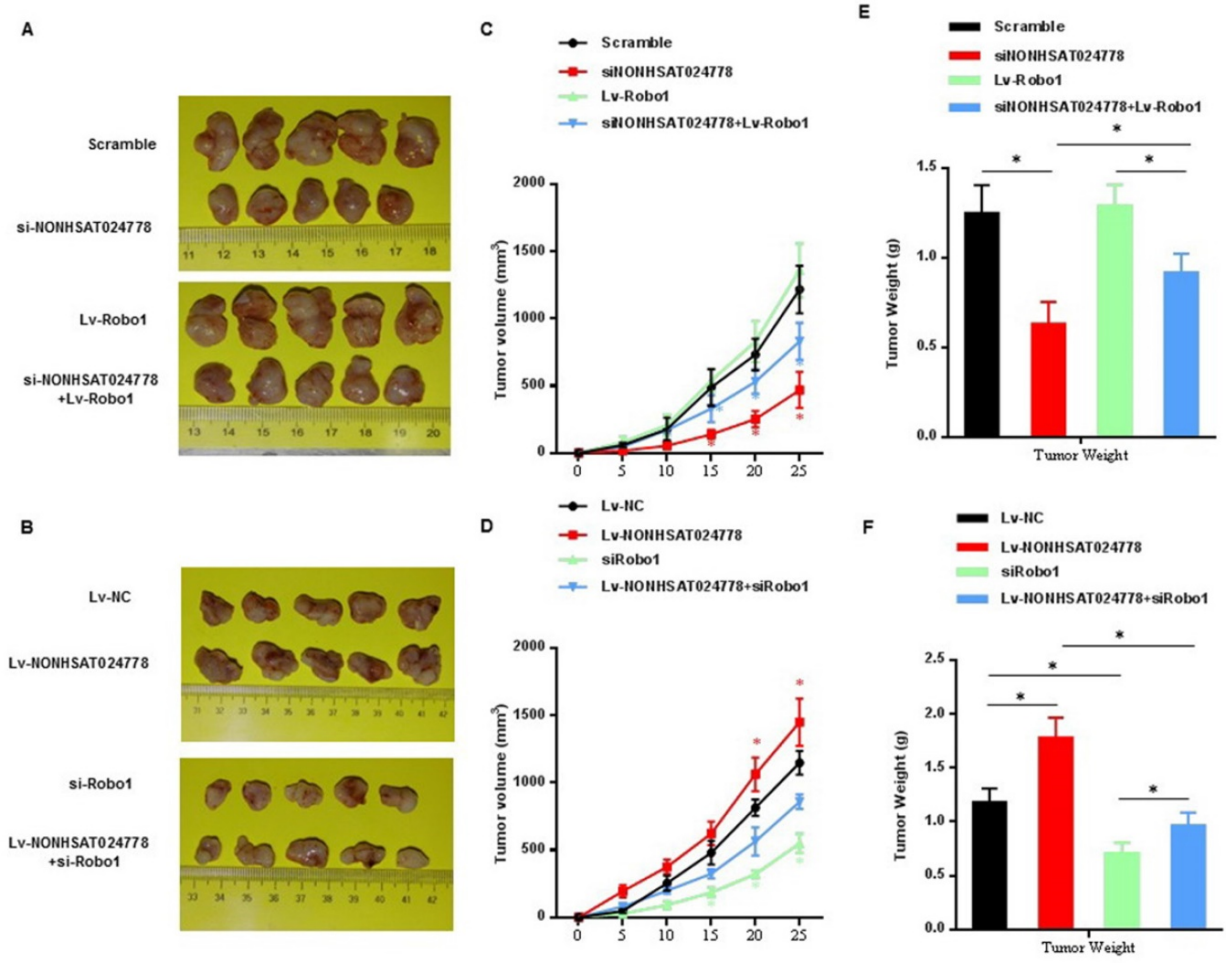
The effects of NONHSAT024778 and Robo1 on tumor growth in a xenograft mouse model (*P<0.05). (A, B) The total visible tumors formed in si-NONHSAT024778, Lv-Robo1, si-NONHSAT024778+Lv-Robo1, Lv-NONHSAT024778, si-Robo1, Lv-NONHSAT024778+si-Robo1, or NC groups of nude mice, respectively. **(C, D)** Growth curves of tumor volume were calculated every 5 days. **(E, F)** Tumor weights from each group are represented.

U-CH1 cells were stably co-transfected with si-NONHSAT024778/Lv-Robo1 or Lv-NONHSAT024778/si-Robo1 and control vector and then were subcutaneously injected into NOD/SCID mice separately to further confirm the effects of NONHSAT024778/miR-1290/Robo1 on chordoma cell growth *in vivo*. NONHSAT024778 knockdown inhibited the chordoma tumor growth *in vivo*, whereas its overexpression increased the tumor growth compared with that in the control (Figures 7A, 7B). In addition, the growth curves of tumor volume and average weight of tumors also indicated the same results (Figures 7C-F). Compared with those from the control cells, the tumor generated by Robo1 re-expression in siNONHSAT024778 cells was significantly larger in size and weight (Figures 7C, 7E), whereas the tumor generated by Robo1 re-inhibition in Lv-NONHSAT024778 cells was significantly smaller in size and weight (Figures 7D, 7F). Therefore, NONHSAT024778 governs chordoma growth by sponging miR-1290 to regulate Robo1 *in vivo*.

## Discussion

ncRNAs play various roles in diverse cancer-related abnormal behaviors of cells, including proliferation, apoptosis, angiogenesis, invasion and metastasis 27, 28. Dysregulation of these lncRNAs and miRNAs frequently occurs in various cancers, where they act as tumor suppressors or oncogenes 29, 30. Here, we aimed to investigate the involvement of NONHSAT024778 and miR-1290 and their biological functions in chordoma.

This study is the first to clarify the biologic function of newly identified lncRNA NONHSAT024778 in chordoma and discover the novel chordoma-associated lncRNA NONHSAT024778, which is significantly up-regulated in chordoma cancer tissues and correlated with malignant progress of chordoma cells. The role of NONHSAT024778 in chordoma cell proliferation, migration, and invasion was further examined. *In vitro* and *in vivo* assays revealed that NONHSAT024778 down-regulation suppressed cell proliferation and tumor growth and reduced cell migration and invasion, whereas its overexpression promoted cell proliferation, migration, and invasion. These findings indicate that NONHSAT024778 has an oncogenic role in chordoma tumorigenesis and could be a potential prognostic indicator for chordoma.

As it is widely known, lncRNA located in the cytoplasm can act as ceRNA to sponge miRNA 31, 32. Our previous research results of miRNA microarray profiling and bioinformatics analysis were used to screen out miR-1290 as a candidate miRNA that targets NONHSAT024778 and to clarify the molecular mechanism underlying NONHSAT024778 contribution to chordoma cell progression. miRNAs are 20-30 nucleotides and another well-known type of ncRNAs. As a member of the miRNA families, miR-1290 is down-regulated in various types of malignant tumors and functions as a tumor suppressor in colon cancer, non-small cell lung cancer, and glioma 33-35. Our data showed significant up-regulation of NONHSAT024778 accompanied by a concomitant decrease in miR-1290 expression in chordoma tissues. Bioinformatics analyses predicted the binding site of NONHSAT024778 in miR-1290, and luciferase reporter assays confirmed that miR-1290 is a novel target of NONHSAT024778. In addition, restoring miR-1290 expression could suppress tumorigenesis in chordoma cells induced by NONHSAT024778. Our findings reveal the importance of the interaction between NONHSAT024778 and miR-1290 in tumorigenesis because NONHSAT024778 exerts an oncogenic behavior partly via sponging miR-1290 in chordoma cells.

According to the ceRNA theory, lncRNA exerts biofunction by interacting with miRNA, and thus suppressing the miRNA target gene that regulates tumor development. Hence, miRNA target is an important part of ceRNA network. Online predicting tools were used to predict the potential target genes of miR-1290 and revealed that Robo1 is one of the miR-1290 targets that have not been previously reported. Robo1 is a member of the roundabout family of receptors involved in various cell processes 36. Based on previous studies, nasopharyngeal cancer progression could be suppressed through Robo1 down-regulation 37, and miR-218 inhibits the migration and invasion of glioma u87 cells through the Robo1 pathway 38. These findings indicate Robo1 might be a prognostic biomarker in chordoma. To date, the expression and function of Robo1 in human chordoma remain unexplored. Bioinformatic analysis and luciferase reporter assays verified that miR-1290 targets Robo1 mRNA at its 3'UTR. Furthermore, rescue experiments determined that the effect of NONHSAT024778 knockdown on the proliferation, migration, and invasion of chordoma cells can be partly reversed by miR-1290 inhibition or Robo1 re-expression in U-CH1 cells. These results imply that NONHSAT024778 promotes chordoma progression by targeting the miR-1290/Robo1 axis.

In our study, miR-1290 expression was negatively correlated with Robo1 and NONHSAT024778, and Robo1 was found to be a direct downstream gene of miR-1290. miR-1290 suppressed the proliferation, migration, and invasion of U-CH1 cells by directly targeting Robo1. Moreover, miR-1290 could bind to NONHSAT024778 and Robo1 via miR-1290 response elements, indicating its possible function as a bridge connecting NONHSAT024778 and Robo1. The specific regulatory role of the NONHSAT024778/miR-1290/Robo1 axis in chordoma must be extensively explored in further studies.

In summary, NONHSAT024778 functions as an oncogene promoting the proliferation, migration, and invasion of chordoma cells, and contributes to chordoma progression by up-regulating Robo1 via sponging miR-1290 *in vitro*. These findings suggest that NONHSAT024778 may be a novel target for the diagnosis and therapeutics of chordoma. However, the number of chordoma tissues studied is limited in this study due to the rare occurrence of chordoma. In the future study, we may use more chordoma tissues and cell lines to investigate the mechanism of recurrence and invasion of chordoma.

## Abbreviations

lncRNA: Long noncoding RNA
miRNAs/miR: MicroRNA
ceRNA: competing endogenous RNA
FNP: fetal nucleus pulposus
ncRNA: noncoding RNA
Robo1: roundabout guidance receptor 1
